# Mathematical Modeling of Uniaxial Mechanical Properties of Collagen Gel Scaffolds for Vascular Tissue Engineering

**DOI:** 10.1155/2015/859416

**Published:** 2015-03-05

**Authors:** Ramiro M. Irastorza, Bernard Drouin, Eugenia Blangino, Diego Mantovani

**Affiliations:** ^1^Instituto de Física de Líquidos y Sistemas Biológicos, CONICET-CCT La Plata, B1900BTE La Plata, Buenos Aires, Argentina; ^2^Instituto de Ingeniería y Agronomía, Universidad Nacional Arturo Jauretche, 1888 Florencio Varela, Buenos Aires, Argentina; ^3^Laboratory for Biomaterials and Bioengineering, Canada Research Chair I, Laval University, Quebec City, QC, Canada G1V 0A6; ^4^Laboratorio de Biomecánica, Departamento de Ingeniería Mecánica, Facultad de Ingeniería, Universidad de Buenos Aires, C1063ACV Buenos Aires, Argentina

## Abstract

Small diameter tissue-engineered arteries improve their mechanical and functional properties when they are mechanically stimulated. Applying a suitable stress and/or strain with or without a cycle to the scaffolds and cells during the culturing process resides in our ability to generate a suitable mechanical model. Collagen gel is one of the most used scaffolds in vascular tissue engineering, mainly because it is the principal constituent of the extracellular matrix for vascular cells in human. The mechanical modeling of such a material is not a trivial task, mainly for its viscoelastic nature. Computational and experimental methods for developing a suitable model for collagen gels are of primary importance for the field. In this research, we focused on mechanical properties of collagen gels under unconfined compression. First, mechanical viscoelastic models are discussed and framed in the control system theory. Second, models are fitted using system identification. Several models are evaluated and two nonlinear models are proposed: Mooney-Rivlin inspired and Hammerstein models. The results suggest that Mooney-Rivlin and Hammerstein models succeed in describing the mechanical behavior of collagen gels for cyclic tests on scaffolds (with best fitting parameters 58.3% and 75.8%, resp.). When Akaike criterion is used, the best is the Mooney-Rivlin inspired model.

## 1. Introduction

In vascular tissue engineering (VTE), bioreactors are used to subdue cells in culture on scaffolds with quasi-physiological conditions during the maturation process that is expected to induce extracellular matrix reorganization and to provide mechanical properties to the regenerated tissue thus leading to the development of functional tissue [1, 2]. Strains can easily be generated and measured in bioreactors but stresses can only be estimated, and this is not a trivial task. Commercial bioreactors can generate and noninvasively measure pressure and diameter of constructs but measurements of stresses can only be estimated by models. One of the most promising approaches for the estimation of the stresses constitutes in generating a mechanical model [3]. Mechanical models are needed not only to identify the degree to which the regenerated tissue will match the physiological tissue [4], but also to provide an estimation of the generated internal stress and its role in growth and development of the constructs [1]. Scaffolds made of synthetic or natural biodegradable materials are generally used, and there is a considerable interest in tailoring mechanical properties of scaffolds to facilitate the cell growth [5]. Among the natural scaffolds, collagen gels have received special attention mainly because they present unmatched biological performances, such as its nontoxicity, low immunogenicity, and antigenicity [6]. In addition to its biological relevance, the collagen is the most abundant protein in native tissues and can be isolated easily in large quantities. Specifically, the main functional feature of collagen is load bearing of tensile force in most tissues where mechanical function is essential such as blood vessels, skin, cartilage, and bones [7, 8]. The development of models of the mechanical behaviour of collagen will also facilitate the optimization of the mechanical properties of the construct by modifying the conditions generated in the bioreactor [4].

Stress and strain present a nonlinear behaviour in most of the biological and bioartificial tissues [9–11]. In particular, viscoelasticity has an important role in these materials, mainly because the entire history of the straining determines the material properties. In addition, there is a dependence of the loading rate in the mechanical response. In the search of mathematical relations between force and displacement Fung proposed the quasilinear viscoelastic model [11]. Nekouzadeh et al. [12] and Nekouzadeh and Genin [4] described the two main limitations of this approach: the accuracy and the computational cost in calibration experiments. Therefore the selection of the nonlinear viscoelastic model and the fitting procedure for a specific tissue can also be a computational challenge.

Although the mechanical model of other scaffolds used in VTE (e.g., standard polyglycolic acid gels) is rather well known [2, 13], for collagen gels, the model describing the mechanical behaviour has much less been studied. Shear and uniaxial extension experiments have been studied by Meghezi et al. [10], using relaxation tests, thus proposing a viscoelastic model, represented by a spring (elastic modulus) and Maxwell elements, associated in parallel. A generalization of Fung's model based on incremental uniaxial tests (step and ramp) where both the spring moduli and time constants vary with strain was also proposed by Pryse et al. [14]. Chandran and Barocas have proposed a review of the mechanical properties of collagen gels [9]. The main conclusion was that the collagen network is actively involved in shear and tensile tests. On the contrary, for compression the mechanisms are not well established yet. Achilli and Mantovani [5] measured the mechanical properties (Young modulus) under confined-compression in a study aimed to gel characterization after preconditioning and assuming a linear model. Chandran and Barocas [9] observed that under step-confined compression (10%), collagen gels exhibited a collapse while, under ramp-compression (0.1%/second), no collapse was observed. They concluded that the gel behaves primarily as a viscoelastic solid, with important damping but negligible relaxation on the time scale of their experiments (1800–2400 seconds).

This work focuses on the mechanical constitutive equations of collagen gels in cyclic compression (loading and unloading). We assumed that the mechanical properties of collagen gels should be evaluated under cyclic testing in order to better mimic the cyclic solicitations imposed by the bioreactor to the vascular construct [15]. Mechanical models are written in the form of a dynamic system to frame the work within the systems control theory [16]. In control engineering, the field of system identification (SI) is devoted to building mathematical models of dynamical systems from measured data [17]. Using SI, we implemented a simple and fast way of achieving constitutive equations describing the behaviour of collagen gels under unconfined compression tests. Results were then interpreted by linear viscoelastic models [11], the Moolin-Rivlin approach [18], and a new polynomial model is finally proposed to describe the mechanical behaviour of collagen gels.

## 2. Material and Methods

### 2.1. Sample Preparation

The protocol used to prepare the gels was previously described in [19]. Briefly, type I collagen is extracted from rat-tail tendons and solubilized in acetic acid solution (0.02 N) at 4 g/L. A collagen solution (2 g/L) is mixed with Dulbecco's modified Eagle medium (DMEM, Gibco, Invitrogen Corporation, Burlington, ON, Canada, 1.1X), NaOH (15 mM), and HEPES (20 mM) in deionized water. This mixture is poured into moulds and left to jellify overnight at 4°C.

### 2.2. Mechanical Tests

**Figure 1:**
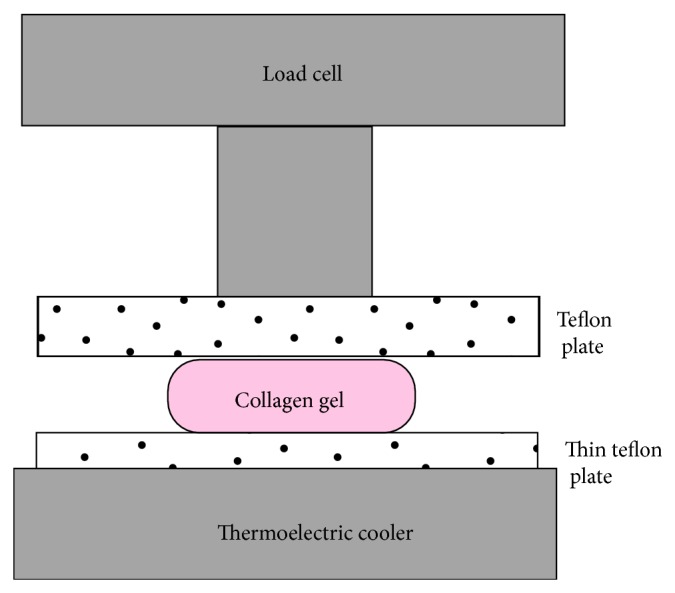
Compression test setup. The teflon plates have a diameter of 70 mm and are attached to a 10 N load cell mounted on an Instron Microtester.

Disk-shaped samples were tested in unconfined compression mode. The 70 mm diameter Teflon compression plate is attached to a 10 N load cell mounted on an Instron Microtester (Instron 5848 Microtester, Instron Corporation, Norwood, MA, USA) as shown in Figure 1. The unconfined compression was performed with a uniform ramp speed ranging from 0.1 to 1 mm/min. The average strain was 20%. Eight cycles (15 to 25% strain) were performed followed by a relaxation test. For this final test, the strain rate is increased to 10 mm/min with a final strain value of 50%. Relaxation tests with final strains of 25% and 50% were also performed with strain rate of 10 mm/min. All tests were performed at 37°C.

### 2.3. Mechanical Models

In this section we will focus on the two proposed mechanical models. Basically, they consist in two Maxwell bodies [11] with nonlinear springs. Two nonlinear springs relationships will be tested: Mooney-Rivlin and polynomial (models 1 and 2, resp.). The general approach is quasi-static, and acceleration effects are neglected. Consequently, the inertia parameter is neglected in the evaluated models.

#### 2.3.1. Model 1

The first model is inspired in the Mooney-Rivlin model, which is applied to uniaxial compression of incompressible and isotropic gels. It leads to a stress-stretch relation written as

(1) $$\begin{array}{c} \sigma =\left(C_{1}+C_{2}\frac{1}{\lambda }\right)\cdot \left(\lambda^{2}-\frac{1}{\lambda }\right), \end{array}$$

where *σ* is the Eulerian stress and *λ* = *e*/*e*_0_ is the stretch ratio (*e* is the extension and *e*_0_ is the extension at instant 0). *C*_1_ and *C*_2_ are constants. Equation (1) yields a linear relationship between *λ* ^−1^ and *σ* · (*λ* ^2^ − 1/*λ*)^−1^. Note also that this becomes the Neo Hookean model when *C*_2_ = 0 [18].

The two Maxwell bodies proposed (with the modified Mooney-Rivlin model) have the next equations:

(2) $$\begin{array}{c} \eta_{1}{\dot{e}}_{1}=g_{1}\left(e^{\prime }-e_{1}\right),\\ \eta_{2}{\dot{e}}_{2}=g_{2}\left(e^{\prime }-e_{2}\right), \end{array}$$

where *e*_*i*_ and *e*′ − *e*_*i*_ are the extension of the dashpot and the spring *i*, respectively. The extension *e*′ is defined as

(3) $$\begin{array}{c} e^{\prime }=\left(C_{1}+C_{2}\frac{1}{\lambda }\right)\cdot \left(\lambda^{2}-\frac{1}{\lambda }\right). \end{array}$$

*g*_*i*_ is the spring constant and *η*_*i*_ the viscosity. Finally, the load is written as follows:

(4) $$\begin{array}{c} TA_{0}=\left[g_{1}\left(e^{\prime }-e_{1}\right)+g_{2}\left(e^{\prime }-e_{2}\right)\right]\frac{1}{\lambda }, \end{array}$$

where *T* = *σ*(*e*_0_/*e*) and *A*_0_ is the cross-section area of the sample. It should be remarked that the extension *e*′ varies with time but does not depend on the derivatives of *λ*. By definition, it is a static function of *λ* thus belonging to a memoryless system. The stretch *λ* = (1 − *ɛ*)^−1^ is also a static function of *ɛ*.

#### 2.3.2. Model 2

A linear representation of two Maxwell bodies is

(5) $$\begin{array}{c} \ddot{\sigma }+\frac{\tau_{1}+\tau_{2}}{\tau_{1}\tau_{2}}\dot{\sigma }+\frac{\sigma }{\tau_{1}\tau_{2}}=\left(g_{1}+g_{2}\right)\ddot{\varepsilon }+\left(\frac{g_{1}}{\tau_{1}}+\frac{g_{2}}{\tau_{2}}\right)\dot{\varepsilon }, \end{array}$$

where *ɛ* = (*e* − *e*_0_)/*e* is the strain, and *τ*_*i*_ = *η*_*i*_/*g*_*i*_ is the relaxation time due to Maxwell body “*i*”. Instead of using *ɛ*, we propose here to use a nonlinear function of the strain, in particular, an order three polynomial function of the strain:

(6) $$\begin{array}{c} f\left(ɛ\right)=p_{0}\varepsilon^{3}+p_{1}\varepsilon^{2}+p_{2}ɛ. \end{array}$$

### 2.4. Nonlinear System Modeling

In this section we will consider the above described viscoelastic models as nonlinear dynamical systems in state space representation. From now on we will denominate *u*, *y*, and *x*_*i*_ as input, output, and state space variable, respectively. All the mentioned variables vary with time.

#### 2.4.1. Model 1

Replacing in (2)–(4) the state space representation variables (*x*_1_ = *e*_1_ and *x*_2_ = *e*_2_) and taking *u* = *e* and *y* = *TA*_0_, a nonlinear system is now defined as

(7) $$\begin{array}{c} {\dot{x}}_{1}=\frac{g_{1}}{\eta_{1}}\left(e^{\prime }-x_{1}\right),\\ {\dot{x}}_{2}=\frac{g_{2}}{\eta_{2}}\left(e^{\prime }-x_{2}\right),\\ y=\left[g_{1}\left(e^{\prime }-x_{1}\right)+g_{2}\left(e^{\prime }-x_{2}\right)\right]\frac{e_{0}}{u}, \end{array}$$

where *e*′ expressed in function of the input of the system is

(8) $$\begin{array}{c} e^{\prime }=\left(C_{1}+C_{2}\frac{e_{0}}{u}\right)\cdot \left[{\left(\frac{u}{e_{0}}\right)}^{2}-\frac{e_{0}}{u}\right]. \end{array}$$

#### 2.4.2. Model 2

**Figure 2:**
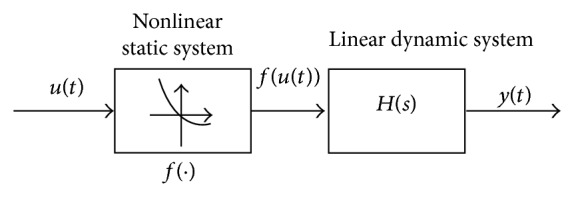
Nonlinear Hammerstein model. The model is defined with a static nonlinear block (the output does not depend on the derivative of the input) followed by a linear time invariant dynamic system.

On the other hand, for implementing the polynomial model we define *u* = *ɛ* and *y* = *σA*_0_, and (5) is modified to

(9) $$\begin{array}{c} \ddot{y}+a_{1}\dot{y}+a_{2}y=b_{0}\ddot{u}+b_{1}\dot{u}, \end{array}$$

where *a*_1_ = (*τ*_1_ + *τ*_2_)/*τ*_1_ *τ*_2_, *a*_2_ = 1/*τ*_1_ *τ*_2_, *b*_0_ = *g*_1_ + *g*_2_, and *b*_1_ = *g*_1_/*τ*_2_ + *g*_2_/*τ*_1_. The state space representation variables are now defined as *x*_1_ = *y* − *b*_0_ *u* and $x_{2}={\dot{x}}_{1}-(b_{1}-a_{1}b_{0})u$, and a state space system is obtained (see [16]):

(10) $$\begin{array}{c} \dot{x}=\left[\begin{array}{cc} 0 & 1\\ -a_{2} & -a_{1} \end{array}\right]x+\left[\begin{array}{c} b_{1}-a_{1}b_{0}\\ -a_{1}\left(b_{1}-a_{1}b_{0}\right)-a_{2}b_{0} \end{array}\right]u,\\ y=\left[\begin{array}{cc} 1 & 0 \end{array}\right]x+b_{0}u, \end{array}$$

where *x*_1_ and *x*_2_ are the elements of the row vector **x**. This is a linear system but if instead of using *u* we use *u*′ = *f*(*u*) = *f*(*ɛ*), a block-oriented Hammerstein nonlinear system is obtained (see Figure 2).

Equations (7) and (10) (using *u*′ as input) constitute the nonlinear systems to be tested in the following sections.

### 2.5. System Identification

The fitting procedure of (7) and (10) to real data is a computational challenge. SI is a well established area of control system theory devoted to find mathematical models from measurements [17, 20, 21]. The basic idea of these techniques is as follows. Let us assume that an input signal *u*(*t*) is applied to a dynamical system for a determined time interval *t*_1_ < *t* < *t*_*N*_. The corresponding output signal is obtained, *y*(*t*). Suppose that both signals are sampled at *t*_*k*_ instants, then data can be denoted by {*u*(*t*_*k*_); *y*(*t*_*k*_)}_*k*=1_^*N*^. A general form (or structure) for the mathematical model is assumed and then the parameters are determined from experimental data. Often, a variety of model structures are evaluated, and the most successful ones are retained. The data are used to build the model by minimizing the difference between the output of the estimated model and the measured output *y*(*t*_*k*_). This minimization can be based on statistical techniques which ensure the robustness of the method. An important step in a SI process is the validation of the model, which must be performed with an independent data set. In this work, we will use one sample gel data set for identification of the model and the remaining samples for validation purposes. One of the validation parameter is

(11) $$\begin{array}{c} \text{FIT}=100\cdot \left(1-\frac{\left|y_{\text{model}}-y_{\mathrm{meas}}\right|}{\left|y_{\mathrm{meas}}-\text{mean}\left(y_{\mathrm{meas}}\right)\right|}\right), \end{array}$$

where *y*_model_ and *y*_meas⁡_ are the output given by the estimated model and the measured data, respectively. It is important to remark that the validation is not a new fitting: the measured input is applied to the identified model and then the obtained output is compared to the measured one. The final prediction error (FPE) is also defined in Appendices as another parameter for evaluation of the model. This parameter is computed with the identification data.

Several tools were developed to implement the SI algorithms. In this paper two Matlab toolboxes are used: System Identification and CON-tinuous-Time System IDentification (CONTSID toolbox [22]).

Particularly in this work, linear and nonlinear SI are tested. For the former,* srivc* algorithm is applied [20] using the structure given by (10) and the strain as input (*u* = *ɛ*). For the nonlinear SI we implement grey-box SI using (7) (model 1) and (10) (model 2, using (6) as input *u*′ = *f*(*ɛ*)). Levenberg-Marquardt algorithm was used to minimize the prediction error of the models.

## 3. Results

### 3.1. Relaxation

**Figure 3:**
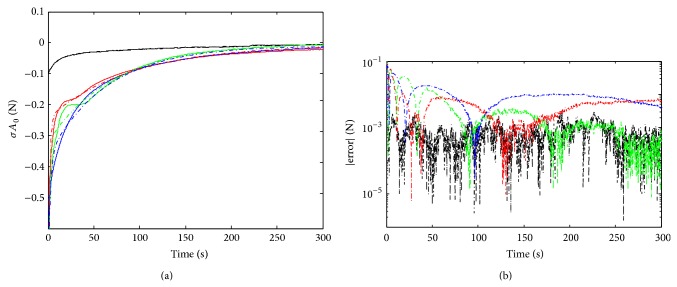
Relaxation tests. (a) Load relaxation in continuous black line corresponds to strain step of 25%. Red, green, and blue continuous lines are the measured relaxations using a step strain of 50%. Both strains are also applied to the estimated models and the values are plotted in dashed-dotted lines with the same color code. (b) shows the time evolution of the absolute error between the experimental curves and models fitting curves in logarithmic scale.

Figure 3 shows the relaxation tests performed at two strain steps, 25% (one sample) and 50% (three samples). Continuous time linear system identification, particularly,* srivc* algorithm was used to obtain transfer functions of the form of (A.1). The input of the system is the step of the strain (*u* = *ɛ* = (*e* − *e*_0_)/*e*) and the output is the load (*y* = *σA*_0_). The best model outputs are also plotted in Figure 3. Curve almost overlaps for the 25% test and the errors (equal to |*y*_model_ − *y*_meas⁡_| which are represented in the inset figure) were always lower than 0.01 for the 50% tests. The dynamics of such identified model and the physical Maxwell bodies time constants (*τ*_*i*_) are related. In fact, the poles of the transfer functions are the inverse of the time constants of the Maxwell bodies. The values obtained for 25% of strain are *τ*_1_ ≈ 11 and *τ*_2_ ≈ 180 seconds and for 50%, *τ*_1_ ≈ 20 and *τ*_2_ ≈ 112 seconds. The results presented here give rise to the proposal of the two Maxwell bodies model for this study.

### 3.2. Cycling

**Figure 4:**
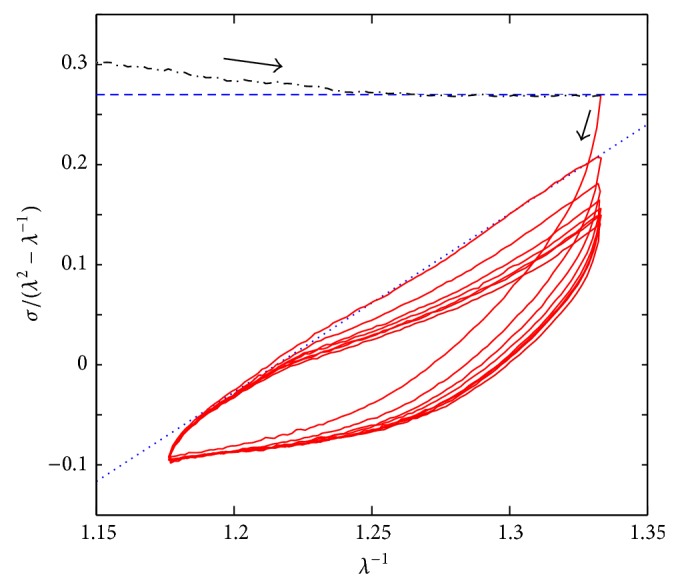
Mechanical behavior of cycling compression test of sample 1. Measured data are plotted in dashed-dotted black line (first compression) and red continuous line. Limit cases are plotted in dashed blue line (Neo Hookean nonviscous model using *C*_2_ = 0, constant value) and in dotted blue line (Mooney-Rivlin nonviscous model). The arrows indicate the time evolution.

Table 1 shows the experimental conditions of the cyclic tests for the four samples. As indicated by (1) in Section 2.3.1, a plot of *λ* ^−1^ versus *σ* · (*λ* ^2^ − 1/*λ*)^−1^ helps in determining whether Mooney-Rivlin model fits some region of the stress-strain behavior. Such a plot in Figure 4 suggests that the first compression (plotted in dashed-dotted black line) can be represented by a Neo Hookean model (which is plotted in dashed blue line). The compression phases of the subsequent cycles (plotted in red line) are well described by Mooney-Rivlin model (plotted in dotted blue line). The hysteresis loop appears because of the cyclic test and is an evidence of a viscous response of collagen gels. These findings give rise to the proposal of the Mooney-Rivlin inspired model described by (7).

**Figure 5:**
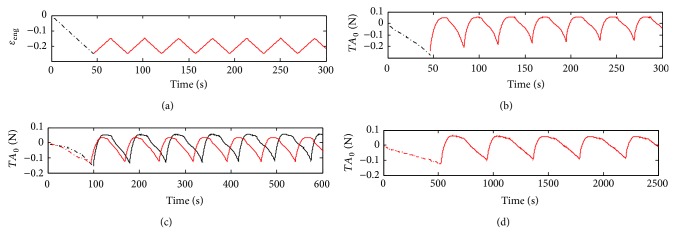
Identification data (sample 1) and validation data (samples 2, 3, and 4). (a) and (b) correspond to engineering strain (*ε*_eng_ = (*e* − *e*_0_)/*e*_0_) and load (*TA*_0_) of sample 1, respectively. (c) correspond to *TA*_0_ of sample 2 (red) and 3 (black). In (d) is shown sample 4. The dashed-dotted lines indicate the first compression of each sample (Neo Hookean behavior).

Identification and validation data are presented in Figure 5. In order to excite all the dynamical modes, the fastest strain data are used for identification which correspond to sample 1.

**Figure 6:**
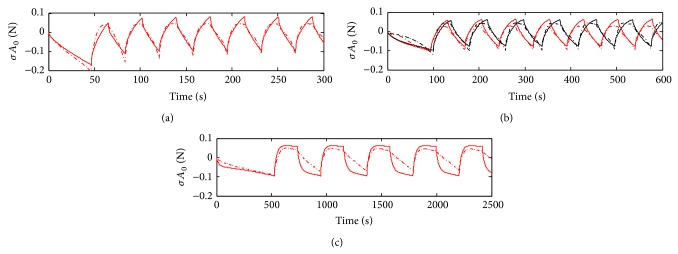
Linear system identification. (a) Identification data using (A.1) and* srivc* algorithm. Measured and estimated data are shown in dashed-dotted and continuous red line, respectively. Validation data of sample 2 (red) and sample 3 (black) are shown in (b), with the same lines code. (c) shows the validation data of sample 4.

The best linear model identified is presented in Figure 6. The model structure is given by (A.1). *u* = *ɛ* (strain) is used as input and *y* = *σA*_0_ as output. The continuous time* srivc* algorithm is used. The parameters obtained are *τ*_1_ ≈ 1 s and *τ*_2_ ≈ 20 s.

**Figure 7:**
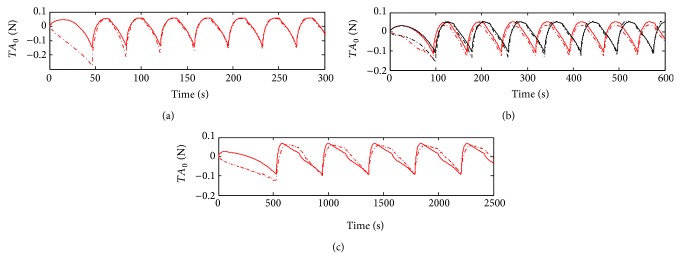
Grey box nonlinear system identification using Mooney-Rivlin inspired model (model 1 with structure given by (7)). (a) Identification data. Measured and estimated data are shown in dashed-dotted and continuous red line, respectively. Validation data of sample 2 (red) and sample 3 (black) are shown in (b), with the same lines code. (c) shows the validation data of sample 4.

Figure 7 shows the results for the Mooney-Rivlin model. Algorithms time constants were the ones identified in the linear cases (*τ*_1_ ≈ 1 s and *τ*_2_ ≈ 20 s). For model 1, the shape of the wave is followed almost superimposed and the estimated parameters are *C*_1_ = −5.61, *C*_2_ = 4.4, *g*_1_ = 0.63, and *g*_2_ = 5.21.

**Figure 8:**
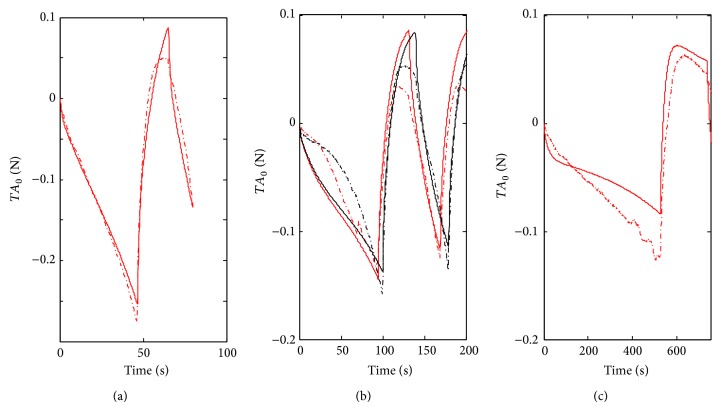
Grey box nonlinear system identification using Neo Hookean inspired model (model 1 with structure given by (7) and *C*_2_ = 0). (a) Identification data. Measured and estimated data are shown in dashed-dotted and continuous red line, respectively. Validation data of sample 2 (red) and sample 3 (black) are shown in (b), with the same lines code. (c) shows the validation data of sample 4.

If the constant *C*_2_ is now fixed to 0, the model 1 is now similar to a Neo Hookean model.  Figure 8 shows the results when the identification of the model is performed with such a model structure. The first compression of sample 1 is well reproduced by the model 1 (with *C*_2_ = 0). This is not the case of the validation data Figures 8(b) and 8(c). The estimated parameters are *C*_1_ = 0.22, *g*_1_ = 5.94, and *g*_2_ = 31.58.

**Figure 9:**
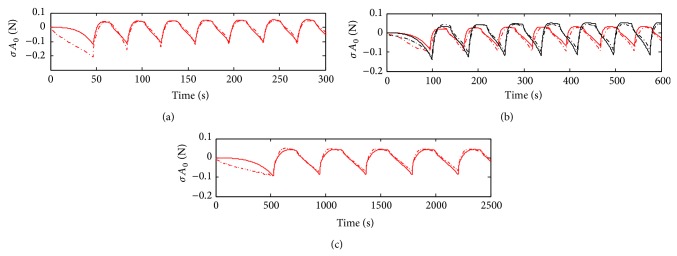
Block-oriented Hammerstein model system identification using a polynomial static function of order three. The structure is given by (10) using *u*′ = *f*(*u*) = *f*(*ɛ*) as input. (a) shows the identification data. (b) and (c) show validation data of samples 2, 3, and 4 using the colors and lines code of Figures 6–8.

The results for the Hammerstein model are presented in Figure 9. The parameters obtained were *p*_*i*_ = 10.8, −1.1 and 0 for *i* = 0,1, and 2, respectively, and also *g*_1_ = 0.81, *g*_2_ = 0.13, *τ*_1_ = 4 s, and *τ*_2_ = 70 s.

The FIT parameters obtained for the best models are presented in Table 2 for the validation data. The Hammerstein model yields the highest FIT parameter: 75.8%. It occurred for sample 3. For sample 4, Hammerstein model also yields a high FIT parameter. Finally, the FPE parameters (computed with identification data at cycling part only) for the linear model, model 1 (Mooney-Rivlin inspired model), and model 2 (Hammerstein with polynomial function of the strain) are 2.89 × 10^−4^, 1.53 × 10^−4^, and 3.13 × 10^−4^, respectively.

## 4. Discussions

This study explored several aspects of the nonlinear relation between uniaxial extension and load for collagen based gels. Compression relaxation and cyclic tests were realised in order to study the mechanical behavior. System identification techniques for processing data were proposed.

Relaxation compression tests indicate that two Maxwell bodies can represent the collagen gel presented here. Regarding the cyclic response of the material, linear Maxwell behavior can not correctly explain the measurements. To interpret it, the analysis of the cyclic tests is divided in two parts: (i) first excursion of the cyclic test (first compression) and (ii) the rest of the cycling.

Figure 8 shows the results of the first excursion (i). According to the identification data, the Neo Hookean inspired model (model 1 using *C*_2_ = 0) seems to represent very well the measurements (Figure 8(a)), but this must be carefully interpreted. It should be noted that the fitting of validation data for the first excursion is not good (Figures 8(b) and 8(c)). Strictly, it can not be concluded that this is the mechanical model of the gels.

For the rest of the cycling data (ii), the linear model (Figure 6) do not represent qualitatively the measurements. However the FIT parameters are surprisingly high. The Mooney-Rivlin inspired model (model 1) has a relatively good FIT parameter, 58.3% for sample 3 (Table 2). If we observe only the second part of the cycling (from 200 to 600 seconds), the fitting is still better (curves almost overlap for samples 2 and 3, see Figure 7(b)). This is not the case of the sample 4; see Figure 7(c). With the best Hammerstein model from 200 to 600 seconds the fitting is even better (see Figure 9(b)). The Hammerstein model achieved the best fitting when it was applied to sample 4: 58.2% (Figure 9(c)). Although Mooney-Rivlin models have very good fitting parameters for this part of the compression test, Hammerstein structure, both quantitatively and qualitatively, yields a better description of the wave form. Considering the FPE parameter the best result is obtained with the Mooney-Rivlin inspired model. This indicates that according to the Akaike criterion the best result is obtained with the model 1.

A commentary should be added regarding the mechanical parameters (*η*_*i*_ and *g*_*i*_) obtained with the presented technique. Although modified two Maxwell bodies model is the proposed model, we cannot strictly say that *η*_*i*_ is the viscosity and *g*_*i*_ is the modulus (note that for model 1 the extension *e*′ depends on *C*_1_ and *C*_2_). However, we can compute *η*_1_ ≈ *C*_2_ *τ*_1_ *g*_1_/*A*_0_, as samples have *A*_0_ ≈ 700 mm^2^; then it yields the value 3.4 kPa s. Using the same reasoning for *η*_2_≈ 570 kPa s. Both values are comparable with the one obtained in [10].

Arteries are complex hierarchical systems, in which three types of cells (endothelial, smooth muscular, and fibroblasts) cohabit in perfect symphony in a sort of basal membrane composed by several types of proteins and biochemical factors all devoted to providing adequate support and specific signals for regulating the overall biological activity and, as a consequence, the mechanical properties [23]. When bioengineers target the regeneration of an artery, by definition, they focused on an engineering approach thus aiming at reproducing a pseudoartery. Cells are often the missed components, although their importance is crucial for their ability to regenerate tissue, organize proteins, and secrete their own extracellular matrix. In this context, computational model can be very effective in providing bioengineers the important factors to consider during experimental approaches.

## 5. Conclusions

The viscoelastic nature of collagen gels under dynamic solicitation is encountered in bioreactors or in mechanobiology experiments. This behavior is particularly hard to anticipate. The tools and models commented on here frame the discussion in terms of system identification methods. This approach introduces engineering concepts, such as the design of experiments and optimal experimental design, to increase the amount of information that can be retrieved from experiments. In future work we plan to study living cells within the collagen gel matrix and to monitor evolution of the mechanical properties using the models presented here.

## Figures and Tables

**Table 1:** Experimental conditions of the cyclic tests.

	Speed	Strain	Purpose
Sample 1	1.0 mm/min	20%	Identification
Sample 2	0.5 mm/min	20%	Validation
Sample 3	0.5 mm/min	20%	Validation
Sample 4	0.1 mm/min	20%	Validation

**Table 2:** FIT parameters of the identified model obtained with the validation data (samples 2, 3, and 4). The methods and algorithms are also detailed. The validation data are referenced to the corresponding figure. M-R, L, and NL mean Mooney-Rivlin, linear and nonlinear.

FIT [%]	*u*	*y*/*A*_0_	System	Structure	Figure
Sample 2					
57.5	*ε*	*σ*	L	Equation (A.1)	Figure 6(b)
37.8	*e*	*T*	NL	M-R	Figure 7(b)
46.9	*ε*	*σ*	NL	Hammerstein	Figure 9(b)
Sample 3					
65.5	*ε*	*σ*	L	Equation (A.1)	Figure 6(b)
58.3	*e*	*T*	NL	M-R	Figure 7(b)
75.8	*ε*	*σ*	NL	Hammerstein	Figure 9(b)
Sample 4					
11.3	*ε*	*σ*	L	Equation (A.1)	Figure 6(c)
21.3	*e*	*T*	NL	M-R	Figure 7(c)
58.2	*ε*	*σ*	NL	Hammerstein	Figure 9(c)

## Appendices

### A. Transfer Function Model

Equation (9) can be transformed into a linear system representation called transfer function. It is obtained by taking the Laplace transform as follows:

(A.1) $$\begin{array}{c} \frac{Y\left(s\right)}{U\left(s\right)}=\frac{\left(g_{1}+g_{2}\right)s^{2}+\left(g_{1}/\tau_{1}+g_{2}/\tau_{2}\right)s}{s^{2}+\left(\left(\tau_{1}+\tau_{2}\right)/\tau_{1}\tau_{2}\right)s+1/\tau_{1}\tau_{2}}=\frac{b_{0}s^{2}+b_{1}s}{s^{2}+a_{1}s+a_{2}}, \end{array}$$

where now *s* = *jω* is the Laplace frequency and *Y*(*s*) and *U*(*s*) are the Laplace transform of *y*(*t*) = *σ*(*t*)*A*_0_ and *u*(*t*) = *ɛ*(*t*), respectively. From (A.1) it can be seen that the dynamic of this system is dominated by the two poles (*g*_1_/*η*_1_ and *g*_2_/*η*_2_) which are the inverse of *τ*_1_ and *τ*_2_.

### B. Validation Parameter

In order to evaluate the estimated models, FIT parameter was calculated. Another parameter is also computed, the Akaike final prediction error (FPE) defined as

(B.1) $$\begin{array}{c} \text{FPE}=V\left(\frac{1+d/N}{1-d/N}\right), \end{array}$$

where *d* is the number of estimated parameters and *N* is the number of estimation (identification) data and *V* is called loss function:

(B.2) $$\begin{array}{c} V=\frac{1}{N}\overset{N}{\underset{k=1}{\sum }}{\left[y_{\text{model}}(t_{k})-y_{\mathrm{meas}}(t_{k})\right]}^{2}. \end{array}$$

The computation is made with the identification data.
